# Profiling of SARS‐CoV‐2 neutralizing antibody‐associated antigenic peptides signature using proteome microarray

**DOI:** 10.1002/mco2.361

**Published:** 2023-09-03

**Authors:** Mingkun Wu, Jiangfeng Liu, Xinming Wang, Xiaomei Zhang, Te Liang, Lan Chen, Tingxuan Huang, Yanan Li, Chang Zheng, Yehong Yang, Jianwei Wang, Xiaobo Yu, Li Guo, Juntao Yang, Lili Ren

**Affiliations:** ^1^ National Health Commission Key Laboratory of Systems Biology of Pathogens and Christophe Mérieux Laboratory Institute of Pathogen Biology Chinese Academy of Medical Sciences & Peking Union Medical College Beijing China; ^2^ State Key Laboratory of Medical Molecular Biology Institute of Basic Medical Sciences Chinese Academy of Medical Sciences & Peking Union Medical College Beijing China; ^3^ State Key Laboratory of Proteomics, Beijing Proteome Research Center National Center for Protein Sciences‐Beijing (PHOENIX Center) Beijing Institute of Lifeomics Beijing China; ^4^ Key Laboratory of Respiratory Disease Pathogenomics Chinese Academy of Medical Sciences and Peking Union Medical College Beijing China

**Keywords:** antigenic peptides, ELISA, neutralizing antibodies, proteome microarray, SARS‐CoV‐2

## Abstract

The profile of antibodies against antigenic epitopes of severe acute respiratory syndrome coronavirus 2 (SARS‐CoV‐2) during neutralizing antibody (NAb) decay has not been clarified. Using a SARS‐CoV‐2 proteome microarray that contained viral antigenic peptides, we analyzed the characteristics of the humoral response in patients with coronavirus disease 19 (COVID‐19) in a longitudinal study. A total of 89 patients were recruited, and 226 plasma samples were serially collected in 2020. In the antigenic peptide microarray, the level of immunoglobulin G (IgG) antibodies against peptides within the S2 subunit (S‐82) and a conserved gene region in variants of interest, open reading frame protein 10 (ORF10‐3), were closely associated with the presence of SARS‐CoV‐2 NAbs. In an independent evaluation cohort of 232 plasma samples collected from 116 COVID‐19 cases in 2020, S82‐IgG titers were higher in NAbs‐positive samples (*p* = 0.002) than in NAbs‐negative samples using enzyme‐linked immunosorbent assay. We further collected 66 plasma samples from another cohort infected by Omicron BA.1 virus in 2022. Compared with the samples with lower S82‐IgG titers, NAb titers were significantly higher in the samples with higher S82‐IgG titers (*p* = 0.04). Our findings provide insights into the understanding of the decay‐associated signatures of SARS‐CoV‐2 NAbs.

## Graphical abstract

We analyzed the characteristics of humoral responses in COVID‐19 patients in a longitudinal study using a SARS‐CoV‐2 proteome microarray containing viral antigenic peptides. We identified two novel peptide‐IgG antibodies against the SARS‐CoV‐2 spike S2 subunit (aa 811‐825, S‐82) and open reading frame protein 10 (aa 21‐38, ORF10‐3) as effective neutralizing antibodies‐associated signatures post‐infection.

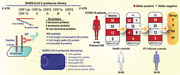

## 1 INTRODUCTION

Severe acute respiratory syndrome coronavirus 2 (SARS‐CoV‐2) has emerged and has been spreading worldwide for more than 3 years.^1^ SARS‐CoV‐2 contains a single‐strand RNA genome that encodes four structural proteins (the spike [S], nucleocapsid [N], membrane [M], and envelope [E] proteins), 16 nonstructural proteins (NSP1−16), and nine accessory proteins (ORF3a‐b, ORF6, ORF7a‐b, ORF8, ORF9b‐c, and ORF10).^2^ Gene site mutations and recombination have generated many SARS‐CoV‐2 variants, and some of the variants have shown antigenic shifts, causing changes in transmissibility and immune escape.^3^, ^4^

The level of neutralizing antibodies (NAbs) that is induced by SARS‐CoV‐2 is critical in evaluating the effects of humoral immunity against viral infections.^5^ The major epitopes that induce NAbs are within the receptor‐binding domain (RBD) and N‐terminal domain of the S protein.^6^, ^7^ The positive correlations between the NAbs titer and factors such as male sex, older age, increased disease severity, high proinflammatory cytokine levels, and especially antibodies binding SARS‐CoV‐2 antigenic proteins have been extensively described.^8^, ^9^, ^10^ Several studies have suggested that the levels of IgG antibodies against SARS‐CoV‐2‐S and N proteins positively correlate with NAb titers.^10^, ^11^, ^12^ The titers of NAbs and IgG antibodies against the SARS‐CoV‐2‐S, N, and RBD proteins decreased during convalescence post‐symptom onset (PSO) and remained detectable at 1 year PSO.^13^ However, the dynamic signature of antibodies against the SARS‐CoV‐2 antigenic epitopes and the correlations with NAbs decay remain unclear.

Proteome microarray is an efficient diagnostic tool for the detection of specific antibodies in infectious and autoimmune diseases.^14^, ^15^ This immunoassay has also been used to analyze B‐cell responses,^16^ antibody dynamics,^17^ and variant‐specific epitopes^18^ in coronavirus disease 2019 (COVID‐19) patients.

In this study, we applied a high‐throughput proteome microarray to characterize the repertoire and kinetics of IgG antibodies against SARS‐CoV‐2 antigenic peptides. The specific peptides related to NAbs seropositivity were also identified and verified. The peptide screening in our study provides candidates for serological assays that can estimate neutralizing capacity post‐SARS‐CoV‐2 infection.

## 2 RESULTS

### 2.1 The construction of the proteome microarray against SARS‐CoV‐2 peptides

**FIGURE 1:**
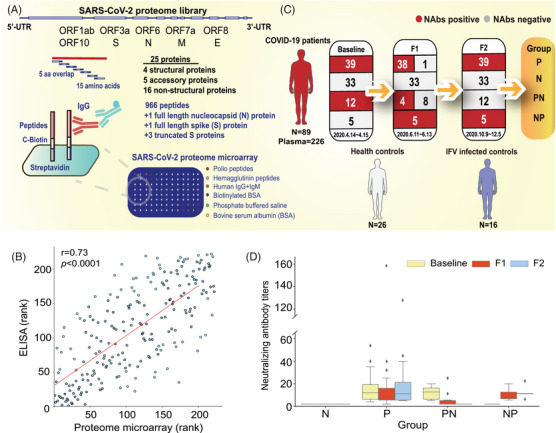
Immunoglobulin G (IgG) antibodies against SARS‐CoV‐2 in recovered patients with COVID‐19 were analyzed using a proteome microarray. (A) Schematic diagram of the proteome microarray used for the detection of SARS‐CoV‐2 peptide‐IgGs. (B) Spearman's correlation for the ELISA and proteome microarray. The *X*‐axis indicates the rank of N‐IgG fluorescence intensity for each sample detected using proteome microarray. The *Y*‐axis indicates the rank of N‐IgG absorbance for each sample detected by ELISA. (C) Research design and participants' information. The plasma samples were collected from patients who had recovered from COVID‐19 during three visits. (D) Neutralizing antibody (NAb) titers against SARS‐CoV‐2 in patients with COVID‐19 during the three visits. The box outlines represent the 25th–75th percentiles and the middle lines indicate the median values. The whiskers indicate 1.5 times the interquartile range (values greater than or lower than the extremes were regarded as outliers). F1, Follow‐up 1; F2, Follow‐up 2; N, NAbs‐negative; P, NAbs‐positive; PN, NAbs‐negative conversion during follow‐up; NP, NAbs‐positive conversion during follow‐up.

To profile the antibodies against SARS‐CoV‐2 antigenic peptides, we constructed a proteome microarray with peptides designed according to the original SARS‐CoV‐2 strain (GenBank MN908947.3), as previously reported.^19^ The peptides used to construct the microarray were 15 amino acids (aa) long with five aa overlap (Figure 1A). The peptides were labeled with a C‐terminal biotin group and printed in duplicate onto a three‐dimensional modified microscope slide using biotin–streptavidin chemistry. Phosphate‐buffered saline (PBS), bovine serum albumin (BSA), and hemagglutinin (HA) peptides were used as negative controls. Biotinylated BSA, human IgG and immunoglobulin M (IgM), and polio peptides were used as positive controls. The lowest limit of detection was 94 pg/mL. The antibody detection efficacy of the proteome microarray was evaluated by comparing it with N‐IgG titers in plasma samples from 222 patients with COVID‐19 that were measured using the ELISA method. A strong positive correlation was observed between the results derived by using the two methods (Spearman *r* = 0.73; *p* < 0.0001), indicating the validity and reproducibility of the proteome microarray (Figure 1B).

### 2.2 Profile of SARS‐CoV‐2 antibodies in plasma samples using proteome microarray

To profile the antibodies in COVID‐19 patients PSO using the proteome microarray, we recruited 89 recovered patients with COVID‐19, and a total of 226 plasma samples were separately collected in 2020 in April (baseline), June (follow‐up 1 or F1), and October–December (follow‐up 2 or F2). The median age of the patients was 47 years (range: 5−80; interquartile range [IQR]: 36−58; Table S1). Three plasma samples were serially collected from 49 patients (Figure S1). Furthermore, 26 samples were collected from 26 healthy individuals (H group) and 16 samples were collected from 16 influenza virus‐infected individuals (IFV group, Figure 1C).

The NAbs titer in each patient was tested using the microneutralization method with cultured virus in our previous report.^20^ The NAbs‐positive rate was 57.3% (51 out of 89) in all patients at baseline, decreased to 52.8% (47 out of 89) at F1, and remained at 49.4% (44 out of 89) at F2. The patients were divided into four groups based on NAbs seropositivity at baseline and F2: NAbs‐negative (N group; *n* = 33), NAbs‐positive (P group, *n* = 39), NAbs‐positive conversion during follow‐up (NP group; *n* = 5), and NAbs‐negative conversion during follow‐up (PN group; *n* = 12; Figures 1C–D). In the PN group, eight individuals were NAbs‐negative at F1, and four were negative at F2 (Figure 1C).

The levels of IgG antibodies against SARS‐CoV‐2 antigen epitopes were tested using the proteome microarray. The fluorescence intensity of IgG in each sample was normalized. We observed that levels of N‐IgG (FDR_baseline, F1, F2_ < 0.0001), S‐IgG (FDR_baseline, F1, F2_ < 0.0001), S1‐IgG (FDR_baseline, F1_ < 0.0001, FDR_F2_ = 0.006), S2‐IgG (FDR_baseline, F1, F2_ < 0.0001), and RBD‐IgG (FDR_baseline, F1, F2_ < 0.0001) were higher in patients who had recovered from COVID‐19 than those in the H and IFV groups during the three visits.

### 2.3 Antibody signatures related to the presence of NAbs in patients who had recovered from COVID‐19

**FIGURE 2:**
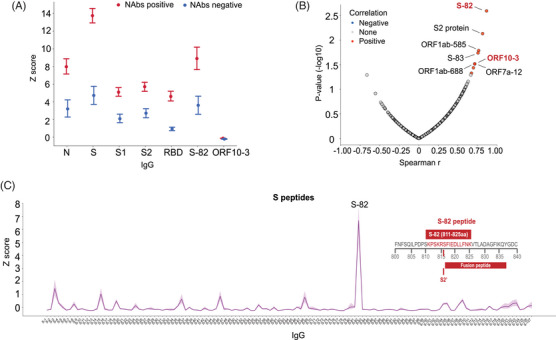
Humoral immune features in patients who had recovered from COVID‐19. (A) The IgG differences between neutralizing antibodies (NAbs)‐positive and NAbs‐negative samples. The middle points indicate the mean *Z* score of each IgG, and the upper and lower lines indicate the 95% confidence interval (CI). (B) The Spearman coefficients of IgG changes and NAbs titer decay in the patients with NAbs‐negative conversion (PN group). (C) The mean *Z* scores of S peptide‐IgG antibodies in all of the samples. S82‐IgG exhibited the highest *Z* score across all S peptide‐IgGs. The amino acid sequence and location of S‐82 are shown in the upper right.

To gain insight into the antibody repertoire related to the presence of NAbs, the IgG levels in NAbs‐positive samples were assessed. We observed that N‐IgG, S‐IgG, S1‐IgG, S2‐IgG, and RBD‐IgG showed higher *Z* scores in NAbs‐positive samples than in negative samples (FDR < 0.0001; Figure 2A). As expected, these IgG antibodies were also dominant (*Z* score > 1.96) in most NAbs‐positive samples but not in NAbs‐negative samples (Table S2).

Two peptide‐IgG antibodies against S‐82 (FDR < 0.0001), and ORF10‐3 (FDR < 0.0001) exhibited higher *Z* scores in NAbs‐positive plasma samples than in NAbs‐negative samples (Figure 2A). Notably, we noticed the positive correlations of S82‐IgG (Spearman *r* = 0.866, *p* = 0.003) and ORF10‐3‐IgG (Spearman *r* = 0.714, *p* = 0.03) with NAbs during follow‐up in the PN group (Figure 2B). These findings supported the close association of the levels of these two peptides with NAb titers. S82‐IgG exhibited the highest mean *Z* score among all of the S‐derived peptides in all of the samples (Figure 2C) and the IgG against this peptide was dominant in 77.9% (102 out of 131) of NAbs‐positive samples (Table S2). Based on logistic regression analysis, the *Z* scores of S82‐IgG (*β* = 0.13, *p* < 0.0001) and ORF10‐3‐IgG (*β* = 4.38, *p* = 0.002) also supported their significant association with the probability of NAbs seropositivity. These findings suggested that S‐82 and ORF10‐3 may be antigenic peptide signatures that indicate SARS‐CoV‐2 NAbs seropositivity following viral infection.

**FIGURE 3:**
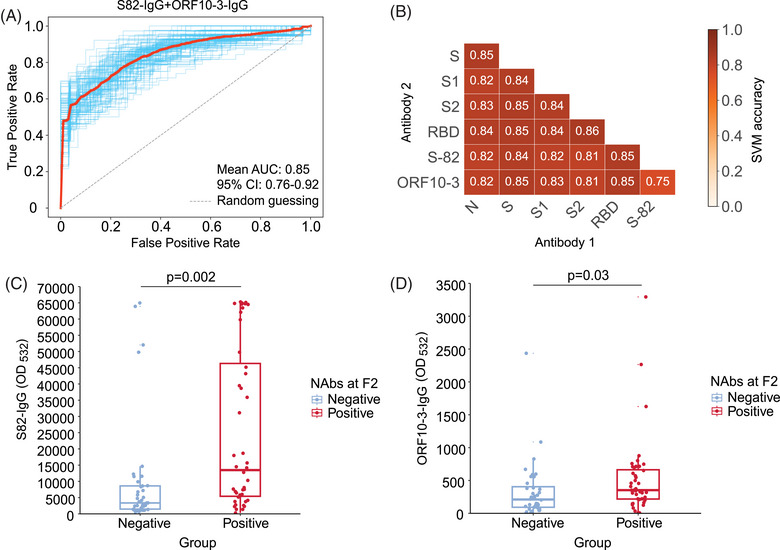
The potency of S‐82 and ORF10‐3 immunoglobulin G (IgG) for SARS‐CoV‐2 neutralizing antibody (NAb) seropositivity evaluation. (A) Receiver operating characteristic (ROC) curve of logistic regression to evaluate NAbs seropositivity in the testing set using S82‐IgG and ORF10‐3‐IgG. The *X*‐axis indicates the false positive rate, and the *Y*‐axis indicates the true positive rate. ROC curves (blue) were generated after 100 runs of computational cross‐validation and the mean ROC curve (red) was generated by averaging all of the ROC curves. The mean and 95% confidence interval (CI) of the areas under the curves (AUCs) after 100 runs of computational cross‐validation were also calculated. (B) The mean accuracy of different IgG combinations for SARS‐CoV‐2 NAbs seropositivity evaluation in support vector machine classifiers with 10‐fold cross‐validation. The two IgGs combined in the classifier were referred to Antibody 1 and Antibody 2. (C and D) The level of IgGs against S‐82 (C) and ORF10‐3 (D) at the last visit before F2 across groups. The dots indicate the IgG absorbance in the proteome microarray. The box outlines represent the 25th–75th percentiles and the middle lines indicate the median values. The whiskers indicate 1.5 times the interquartile range (values greater than or lower than the extremes were regarded as outliers). The *p* values in the Kolmogorov–Smirnov test are shown. OD_532_ = fluorescent signal intensity at 532 nm.

We next evaluated the effect of S‐82 and ORF10‐3 as NAbs‐associated peptide signatures. According to the logistic regression's receiver operating characteristic curve, the mean area under the curve (AUC) was 0.85 (95% confidence interval [CI]: 0.76−0.92) when the two IgGs were combined (Figure 3A). The ROC curves achieved AUCs of 0.85 (95% CI: 0.80−0.90) and 0.76 (95% CI: 0.70−0.82) for S82‐IgG and ORF10‐3‐IgG, respectively (Figure S2). Moreover, we trained support vector machine (SVM) classifiers with 10‐fold cross‐validation to evaluate their effectiveness. As shown in Figure 3B, the mean predictive accuracy was 0.75 when S82‐IgG and ORF10‐3‐IgG were combined, and the mean accuracy increased to over 0.80 when S82‐IgG or ORF10‐3‐IgG was combined with N‐IgG, S‐IgG, S1‐IgG, S2‐IgG, or RBD‐IgG.

Based on the proteome data before F2, we observed that the levels of S82‐IgG (Figure 3C; *p* = 0.002) and ORF10‐3‐IgG (Figure 3D; *p* = 0.03) were lower in patients who were NAbs‐negative at F2 than in NAbs‐positive patients during follow‐ups, suggesting that the detection of the two IgGs were a potential early warning signal for NAbs‐negative conversion. Therefore, S82‐IgG and ORF10‐3‐IgG levels may contribute to identifying the presence of NAbs following viral infection.

### 2.4 Kinetics of SARS‐CoV‐2 antigenic peptides associated with NAbs

**FIGURE 4:**
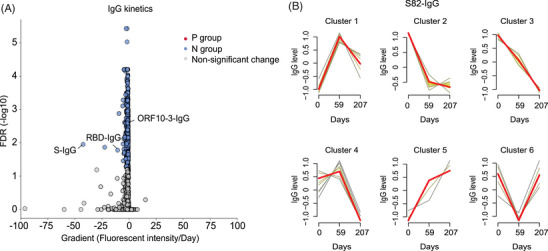
Immunoglobulin G (IgG) kinetics in patients who had recovered from COVID‐19. (A) The decline rates of IgG antibodies in patients with COVID‐19. The *X*‐axis indicates the gradient of the linear mixed‐effects model and represents the fluorescence intensity decline per day. The *Y*‐axis indicates the false discovery rate (−log10). (B) Fuzzy c‐means clustering of S82‐IgG kinetics in patients with COVID‐19. Clusters 1−6 represent the different S82‐IgG kinetics patterns after clustering. The colors represent the membership values of the lines in each cluster, which gradually change from grey to red. The *X*‐axis indicates the median day at each visit, and the median day at baseline refers to Day 0. The *Y*‐axis indicates normalized intensity at each visit.

A total of 49 patients were sampled three times in our study, allowing us to perform longitudinal observation of the IgG antibodies against SARS‐CoV‐2 peptides and structural proteins. Linear mixed‐effect models showed that the levels of IgG antibodies against 349 peptides declined significantly in the N group, including S‐IgG (FDR = 0.01) and RBD‐IgG (FDR = 0.02) (Figure 4A). The IgGs against structural proteins (S, N, S1, S2, and RBD) showed non‐significant changes in the P group (Table S3). Given that the kinetics of IgGs against S‐82 and ORF10‐3 may impact NAbs seropositivity during convalescence, we focused on the dynamics of these IgGs in patients. ORF10‐3‐IgG declined significantly in the N group (FDR = 0.003) but not in the P group (FDR = 0.17) (Figure 4A). The decline of S82‐IgG was non‐significant in the P group (FDR = 0.65) and N group (FDR = 0.34) during the follow‐ups (Table S3). However, by using fuzzy c‐means clustering, we found that the kinetics of S82‐IgG in the patients belonging to the N group and PN group were mainly observed in clusters 2, 3, and 4, all of which exhibited significantly declining trends in IgG levels in serially collected plasma samples (Figure 4B). Our findings suggested that the kinetics of S82‐IgG and ORF10‐3‐IgG were stable with the persistence of NAbs post‐viral infection.

### 2.5 Correlation of SARS‐CoV‐2 S82‐IgG levels with NAb titers

**FIGURE 5:**
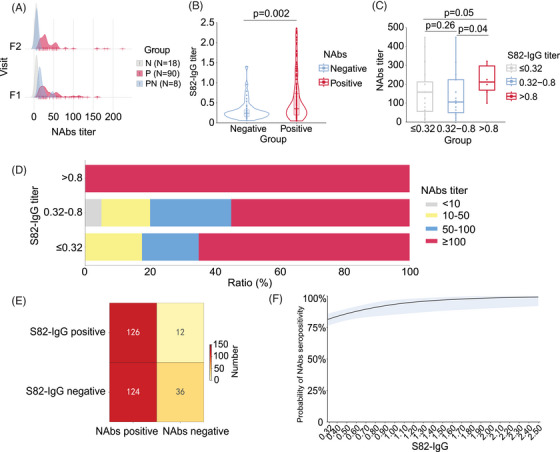
The association of S82‐IgG and neutralizing antibodies (NAbs) in ELISA validation. (A) The NAb titers from an independent evaluation cohort involving 232 plasma samples collected from 116 COVID‐19 cases in 2020. N group, NAbs‐negative patients; P group, NAbs‐positive patients; PN group, patients with NAbs‐negative conversion. (B) S82‐IgG titers in NAbs‐positive samples versus NAbs‐negative samples. The dots in the violin plot indicate the S82‐IgG titers in ELISA and the middle line represents the median value. The *p* value in the Mann–Whitney *U* test is shown. (C) The titers of NAbs against Omicron BA.1 subvariant in the groups with differing S82‐IgG titers. The dots indicate the titers of NAbs against Omicron BA.1 subvariant. The box outlines represent the 25th–75th percentiles and the middle lines indicate the median values. The whiskers indicate 1.5 times the interquartile range (values greater than or lower than the extremes were regarded as outliers). The *p* values in the Mann–Whitney *U* tests are shown. (D) The ratio of individuals with different titers of NAbs against Omicron BA.1 subvariant in the groups with differing S82‐IgG titers. (E) The number of NAbs‐positive and NAbs‐negative plasma samples (*N* = 298) across the groups based on S82‐IgG seropositivity in two independent evaluation cohorts using ELISA. (F) The logistic regression for S82‐IgG titer and the probability of NAbs seropositivity in plasma samples (*N* = 298) from two independent evaluation cohorts. The *X*‐axis indicates the S82‐IgG titer tested using ELISA. The *Y*‐axis indicates the probability of NAbs seropositivity.

To verify the efficacy of S82‐IgG on NAb titers, we evaluated S82‐IgG levels in two independent evaluation cohorts using ELISA. One cohort included 232 plasma samples collected from 116 recovered COVID‐19 patients in June–September 2020 (F1) and December 2020–January 2021 (F2), and 66 plasma samples collected from healthy individuals. NAbs were positive in 98 patients (84.5%) at F1, and 90 patients remained positive (P group), while eight patients were negative at F2 (PN group). Eighteen patients (15.5%) were NAbs‐negative (N group; Figure 5A). As expected, the S82‐IgG titers were higher in NAbs‐positive plasma samples than in NAbs‐negative plasma samples (*p* = 0.002; Figure 5B).

Another cohort included 66 individuals recruited in February 2022 who were vaccinated with inactivated SARS‐CoV‐2 vaccine and then infected with Omicron BA.1 virus (Figure S3). A total of 62 (93.9%) plasma samples were NAbs‐positive against Omicron BA.1. The presence of S82‐IgG was tested using ELISA; among the 26 positive plasma samples, 25 (96.2%) samples were NAbs‐positive. The NAb titers were significantly higher in the samples where the S82‐IgG titers were over 0.80 than those with titers between 0.32 and 0.80 (*p* = 0.04; Figure 5C). Furthermore, NAb titers were higher than or equal to 100 in 55% (11 out of 20) of the plasma samples in which the S82‐IgG titer was between 0.32 and 0.80, and this ratio increased to 100% (six out of six) in the samples with S82‐IgG titers over 0.80 (Figure 5D).

In all of the 298 plasma samples from the two evaluation cohorts, the chi‐square test indicated that NAbs seropositivity was significantly higher in the S82‐IgG positive samples (*χ* ^2^ = 10.45, *p* = 0.001; Figure 5E). The sensitivity of S82‐IgG for NAbs seropositivity evaluation was 50.4% and the specificity was 75% (Figure 5E). Moreover, logistic regression analysis showed a positive correlation between the S82‐IgG titer and the probability of NAbs seropositivity (*β* = 1.63, *p* = 0.008; Figure 5F). These results suggested the correlation of SARS‐CoV‐2 S82‐IgG levels with NAb titers, a finding consistent with that of the proteome assay in our study. In summary, our findings confirmed that S82‐IgG levels can reflect the NAbs‐associated immune signature.

## 3 DISCUSSION

In this study, we profiled antibody signatures post‐SARS‐CoV‐2 infection at the amino acid resolution level by using a proteome microarray containing 966 SARS‐CoV‐2 antigenic peptides.^19^ We observed that the levels of IgG antibodies against structural proteins (S, N, S1, S2, and the RBD) in NAbs‐positive plasma increased significantly compared with those in NAbs‐negative plasma, suggesting higher immunogenicities of these antigens in NAbs‐positive patients. Furthermore, two novel IgG antibodies against antigenic peptides in the S (S‐82, 811−825 aa) and ORF10 (ORF10‐3, 21−38 aa) proteins also exhibited strong associations with NAb titers and are potential early warning signals of SARS‐CoV‐2 NAbs decay.

The proteome microarray has been used to depict the epitope landscape of the SARS‐CoV‐2 spike protein in COVID‐19 patients.^21^ Significant decreases in the IgG response against several epitopes (S1‐93, S1‐97, and S2‐78) in non‐survivor patients were observed, indicating the protective roles of the corresponding antibodies.^22^ In another study, longitudinal and proteome‐wide analyses were used to conclude that the levels of S82‐IgG differed significantly between mild and severe COVID‐19 patients.^23^ In our study, we applied the SARS‐CoV‐2 proteome microarray to profile the specific epitopes related to NAbs decay during convalescence. As expected, strong humoral responses against SARS‐CoV‐2 structural proteins were associated with the presence of NAbs. Notably, we also identified two antigenic peptides (S‐82 and ORF10‐3) that can be used to evaluate NAbs seropositivity and decay. This finding revealed an important role of the S‐82 peptide in reflecting the NAb response post‐SARS‐CoV‐2 infection. NAb titers have been shown to increase over time in parallel with the rise in IgG antibody levels.^9^ However, the effective signatures of humoral responses that could allow the evaluation of SARS‐CoV‐2 NAbs seropositivity post‐infection have remained unclear. The levels of IgG against structural proteins (S1, N, and the RBD) have shown different predictive effects on NAb titers post‐infection and vaccination.^8^, ^24^ Our study revealed that the combination of S82‐IgG and ORF10‐3‐IgG effectively estimated NAbs decay during follow‐ups, providing insight into the relationship between these antigenic peptides and neutralizing capacity and durability against SARS‐CoV‐2. Moreover, longitudinal observation revealed a stable relationship between S82‐IgG and ORF10‐3‐IgG with the persistence of NAbs. These findings suggested the stable role of the peptide‐IgGs in evaluating NAbs seropositivity.

The IgM antibody against S‐82 has been reported to be enriched in patients with COVID‐19.^25^ This 15‐mer peptide is located within the fusion peptide (FP) domain, which is partially exposed on the surface of the spike protein, including in SARS‐CoV‐2 variants of concern.^26^ The conserved residue was observed to be engaged in electrostatic interactions with several FP‐specific monoclonal antibodies (VN01H1 and C77G12) that exhibited heterogeneous neutralizing activities and reduced viral burden in vivo.^27^ Moreover, two broad‐spectrum NAbs (COV44‐62 and COV44‐79) that could neutralize alpha and beta coronaviruses including the Omicron BA.1 and BA.2 subvariants were mapped to the S2' cleavage site using a surface plasmon resonance‐based high‐throughput peptide array.^28^ A previous report suggested that mice immunized with a peptide that included the S2'site and the FP region (aa 816−826) did not exhibit significant neutralization activity.^22^ The positive correlation between the level of S82‐IgG and the NAbs titer observed in our study indicated the potential neutralizing capacity induced by this peptide. Moreover, the decline of IgG levels against this antigenic peptide may reflect the NAbs decay observed during follow‐up visits.

Our study had some limitations. First, the sample size was limited; therefore, the effectiveness of S82‐IgG and ORF10‐3‐IgG in the evaluation of NAbs seropositivity should be confirmed with a large and long cohort study of SARS‐CoV‐2 variants of concern. Second, an ELISA for ORF10‐3‐IgG was not performed because of ORF10‐3‐IgG's high hydrophobicity; therefore, the efficacy of ELISA in the analysis of NAbs must be investigated further. Third, the sensitivity and specificity of the proteome microarray should be confirmed given that the microarray contained only linear epitopes; further studies are needed to investigate the neutralization potential of SARS‐CoV‐2 conformational epitopes.

## 4 CONCLUSION

In this study, we used a proteome microarray to characterize IgG antibody signatures against antigenic epitopes post‐SARS‐CoV‐2 infection. We observed robust humoral responses against SARS‐CoV‐2 structural proteins and antigenic domains after viral infection. Furthermore, IgG kinetics after infection were correlated with epitope sites and NAb titers. We identified two novel peptide‐IgG antibodies against the SARS‐CoV‐2 spike S2 subunit—S‐82 (aa 811−825) and ORF10‐3 (aa 21−38)—as effective NAbs‐associated immune signatures. Our study provides insights into the humoral immune response against SARS‐CoV‐2 infection and proposes novel candidates for the evaluation of SARS‐CoV‐2 NAbs.

## 5 MATERIALS AND METHODS

### 5.1 Participants and samples

The plasma samples were collected from Wuhan, China. The sampling procedure, during which SARS‐CoV‐2 infections were diagnosed in accordance with the Chinese clinical guidance for COVID‐19 pneumonia diagnosis and treatment,^29^ has been described in our previous report on viral seroprevalence.^20^ Demographic information for all of the participants is shown in Table S1. The patients were diagnosed with symptomatic or asymptomatic infection according to whether they had a self‐reported fever or respiratory symptoms (including but not limited to cough, anhelation, stuffy nose, rhinorrhea, sore throat, pneumonia, or both) during clinical care. The plasma samples from all patients were collected on April 14−15 (baseline), June 11−13 (follow‐up 1 or F1), and October 9–December 5, 2020 (follow‐up 2 or F2), when participants were recruited and venous blood samples were collected. Sixteen plasma samples collected from 16 healthy volunteers and 26 plasma samples collected from 26 individuals who recovered from influenza virus infection were used as controls (Table S4).

Two validation cohorts were involved. As the test cohort for S82‐IgG ELISAs, we recruited 116 individuals who had recovered from confirmed COVID‐19 in Wuhan, China. The median age in the test cohort of patients with COVID‐19 was 57 years (range: 28−95; IQR: 49−65; Table S5). Two paired plasma samples (total = 232) were collected from each patient in June–September 2020 (F1) and December 2020–January 2021 (F2). The number of SARS‐CoV‐2 NAbs‐positive samples was 188, and the number of negative samples was 44. We also collected 66 plasma samples from 66 additional healthy individuals in Wuhan (Table S5).

Another validation cohort included 66 individuals who had been vaccinated with inactivated SARS‐CoV‐2 vaccine and then infected with Omicron BA.1 virus in Tianjin, China. The median age of these cases was 40 years (range: 18−69; IQR: 33−56; Table S6). In total, 66 plasma samples were collected from patients on February 11−23, 2022.

All plasma samples were inactivated at 56°C for 30 min before use. Recombinant N protein was used to evaluate the antibodies against SARS‐CoV‐2. NAb titers were assessed at each visit using in‐house microneutralization assays for the original SARS‐CoV‐2 strain or the Omicron BA.1 strain.^20^

### 5.2 Preparation of a SARS‐CoV‐2 proteome microarray

The SARS‐CoV‐2 proteome microarray that contained 966 tiled peptides for each of the N, S, E, M, ORF1ab, ORF3a, ORF6, ORF7a, ORF8, and ORF10 proteins of SARS‐CoV‐2 (GenBank: MN908947.3) was prepared as previously described.^19^ The 15‐mer biotin‐labeled peptides with five overlapping amino acid residues were synthesized (Chinese Peptides, Hangzhou, China; Guoping Pharmaceutical). SARS‐CoV‐2 proteins, including S (Val16–Pro1213), S1 (Val16‐Arg685), S2 (Ser686‐Pro1213), RBD (Arg319‐Phe541), and N (Met1‐Ala419), were expressed in insect cells or human HEK293 cells (Sino Biological). These peptides and proteins were printed onto a three‐dimensional modified slide surface (Capital Biochip Corp) in parallel and duplicated using an Arrayjet microarrayer (Arrayjet). PBS, BSA (100 μg/mL; Sigma–Aldrich), and HA peptides (500 μg/mL; Chinese Peptides) were used as negative controls. Biotinylated BSA (100 μg/mL), human IgG and IgM (10 μg/mL), and polio peptides (500 μg/mL; Chinese Peptides) were used as positive controls. The peptide microarrays were stored at −20°C until they were ready to use.

### 5.3 Detection of viral antibodies using a SARS‐CoV‐2 proteome microarray

The peptide microarrays were assembled in an incubation tray and blocked with 5% (weight‐bulk ratio) milk/(PBS with 0.2% [volume ratio] Tween‐20 or PBST) for 1 min at room temperature. After being washed three times with PBST, the array was incubated with plasma at a dilution of 1/300 for 30 min at room temperature. The microarray was then incubated for 30 min with a mixture containing Cy3 AffiniPure donkey anti‐human IgG (H+L) and Alexa Fluor 647 AffiniPure goat anti‐human IgM FC5 μ antibody (2 μg/mL; both from Jackson ImmunoResearch). Finally, the array was washed with PBST and water, dissembled from the tray, and dried using centrifugation for 2 min at 2000 rpm. The array was scanned with a GenePix 4300A microarray scanner (Molecular Devices) at 10 μm resolution using a laser at 532 nm with 100% power/PMT Gain 800 for IgG. The median fluorescent signal intensity with background subtraction was extracted using GenePix Pro7 software (Molecular Devices).

### 5.4 Enzyme‐linked immunosorbent assay

The 96‐well microplates (Corning, NY, USA) were coated with 200 ng of S‐82 peptide (TGpeptides) and incubated overnight at 4°C. The plates were washed once with PBST buffer and then blocked with 5% BSA for 2 h at 37°C. Samples were diluted to 1/100 with 0.5% BSA and incubated for 1 h at 37°C. After washing with PBST, anti‐human IgG‐peroxidase (Jackson ImmunoResearch) was diluted to 1/60,000 and incubated at 37°C for 1 h. The plates were washed and developed with 100 μL of substrate solution (Solarbio). Finally, 50 μL of stop buffer (Solarbio) was added to stop the reaction. Optical density was detected at 450 nm using a multifunctional microplate reader SpectraMax M5 (Molecular Devices). In the evaluation cohort, the cutoff values were determined by calculating the mean absorbance at 450 nm of the negative control (0.17) and adding three times the standard deviation value (0.15) for healthy individuals, which was 0.32 for S82‐IgG.

### 5.5 Statistical analysis

Each sample's raw fluorescent signal intensity in the proteome microarray was normalized to a *Z* score, and an IgG with a *Z* score over 1.96 was defined as a “dominant IgG” in each sample. The *Z* score of each IgG was compared using the Kolmogorov–Smirnov test. The comparisons of antibody titers in ELISA were performed using the Mann–Whitney *U* test, and the false discovery rate (FDR) in multiple tests was adjusted using the Benjamini–Hochberg approach.

In logistic regression, ln($\frac{p}{1-p}$) = *β*0 + *β*_1_ *x*_1_ + *β*_2_ *x*_2_……, where *p* is the probability of SARS‐CoV‐2 NAbs seropositivity, *x* is the fluorescence intensity of each IgG in the proteome microarray, and *β*0 refers to an intercept. The ratio of the training set to the testing set in the logistic regression was 7:3 when evaluating the predictive effect, and 100 runs of computational cross‐validation were performed. We used the fluorescence intensities of IgG to train SVM classifiers. The linear kernel function was adopted and the penalty factor *C*‐value was set to 1. To examine the stabilities of our classifiers, we performed 10‐fold cross‐validations and calculated the mean accuracy.

To characterize the kinetic differences among patients with COVID‐19, we fitted the following linear mixed‐effects models using paired samples in the proteome microarray: IgG fluorescence intensity ∼ Time + (1 + Time | Patient), and the median day at baseline was referred to as Day 0.

The statistical tests were performed using the Python 3.7 package Statsmodels v0.11.1, and the probability of type I error (α) was set to 0.05. The IgG kinetics were clustered using the R 4.1.2 package Mfuzz v2.58.0, and the number of clusters was set to 6. Visualization of the statistical analysis was achieved using the Python 3.7 packages Matplotlib v3.4.2 and Seaborn v0.11.0, and the R 4.1.2 packages ggplot2 v3.4.2 and Mfuzz v2.58.0.

## AUTHOR CONTRIBUTION

J. Y., X. Y., L. R., and J. W. conceived the idea and designed the experiment. L. C. and X. W. collected the samples. X. Z., T. L., and X. Y. prepared the proteome microarray. J. L., C. Z., L. G., and M. W. performed the quality control of the proteome microarray data and analyzed the proteome microarray data. M. W., Y. L., and T. H. performed the evaluation test. M. W., L. G., and L. R. wrote the manuscript. Y. Y., L. G., L. R., J. Y., and J. W. reviewed the manuscript. All authors have read and approved the final manuscript.

## CONFLICT OF INTEREST STATEMENT

The authors have declared that no competing interests exist.

## ETHICS STATEMENT

This study was approved by the Ethical Review Board of the Institute of Pathogen Biology, Chinese Academy of Medical Sciences (IPB‐2020‐04). Written informed consent was obtained from each enrolled patient.

## Supporting information

Supporting Information

Supporting Information

Supporting Information

Supporting Information

## Data Availability

The data from this study are available in the supplementary materials.
