# Supplementary material for: Profiling of SARS‐CoV‐2 neutralizing antibody‐associated antigenic peptides signature using proteome microarray
Source: MedComm (2020). 2023 Sep 3;4(5):e361. doi: 10.1002/mco2.361 (PMC10475218; doi:10.1002/mco2.361)
Supplement: Supplementary file 1 — Supporting Information [file MCO2-4-e361-s004.docx]

**Profiling of SARS-CoV-2 neutralizing antibody-associated antigenic peptides signature using proteome microarray**

Mingkun Wu^1#^, Jiangfeng Liu^2#^, Xinming Wang^1#^, Xiaomei Zhang^3^, Te Liang^3^, Lan Chen^1^, Tingxuan Huang^1^, Yanan Li^1^, Chang Zheng^3^, Yehong Yang^2^, Jianwei Wang^1,4^, Xiaobo Yu^3*^, Li Guo^1,4*,^ Juntao Yang^2*^, Lili Ren^1,4*^

1. National Health Commission Key Laboratory of Systems Biology of Pathogens and Christophe Mérieux Laboratory, Institute of Pathogen Biology, Chinese Academy of Medical Sciences & Peking Union Medical College, Beijing, China.
2. State Key Laboratory of Medical Molecular Biology, Institute of Basic Medical Sciences, Chinese Academy of Medical Sciences & Peking Union Medical College, Beijing, China.
3. Beijing Proteome Research Center, National Center for Protein Sciences-Beijing (PHOENIX Center), Beijing Institute of Lifeomics, Beijing, China.
4. Key Laboratory of Respiratory Disease Pathogenomics, Chinese Academy of Medical Sciences and Peking Union Medical College, Beijing, China.

* Equal contribution as co-first authors

† Equal contribution as co-senior authors

**Correspondence:**

Xiaobo Yu (yuxiaobo@ncpsb.org.cn) , Li Guo (gnyny0803@163.com), Juntao Yang (yangjt@pumc.edu.cn) , and Lili Ren (renliliipb@163.com)

**Supplementary information materials**

**Table S1.** **Demographic characteristics of participants**


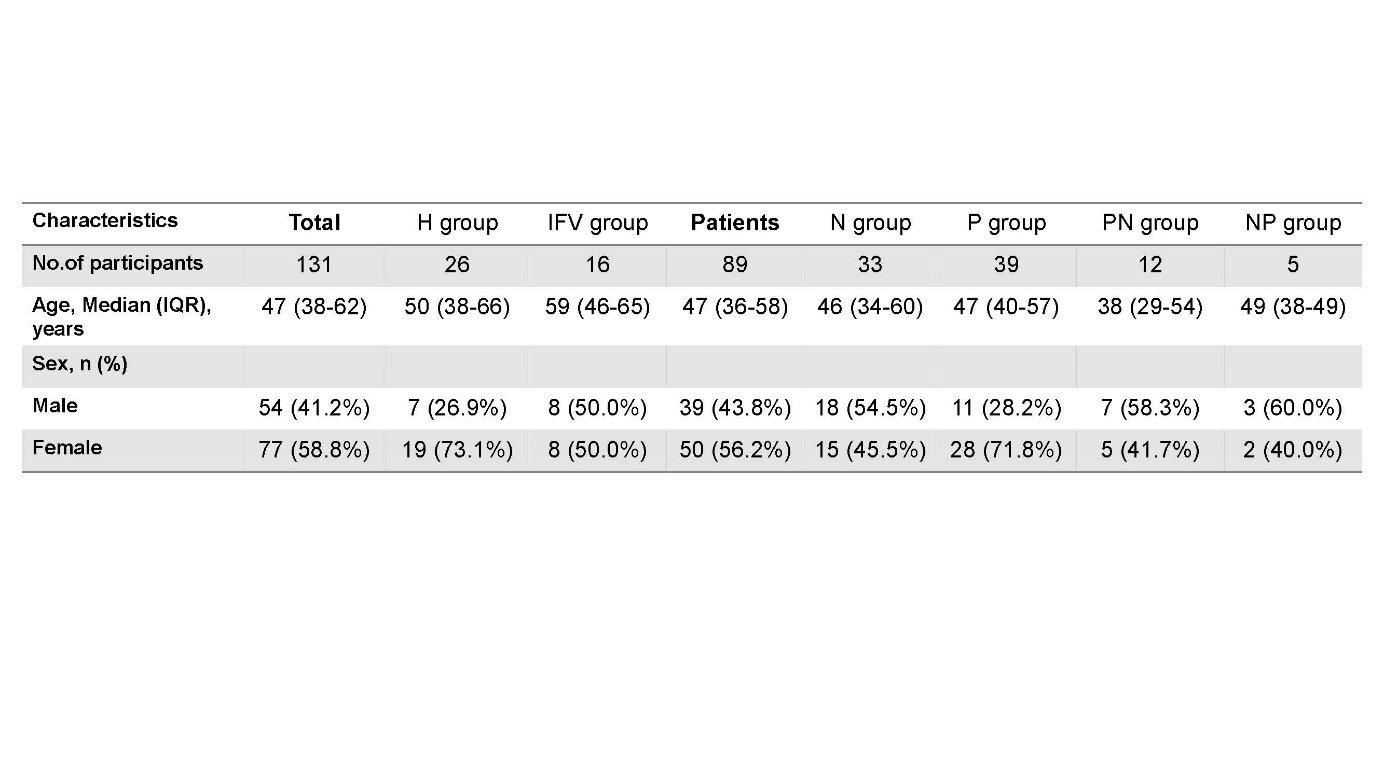


IQR: Interquartile range

**Table S2. Statistical information of dominant IgG (N, S, S1, S2, RBD, and S-82) in all samples**

| IgG | Sample | Numbers (Z score>1.96) | Percentage |
| --- | --- | --- | --- |
| N-IgG | Health | 4/26 | 15.4% |
|  | IFV | 0/16 | 0 |
|  | NAbs Negative | 45/95 | 47.4% |
|  | NAbs Positive | 121/131 | 92.4% |
| S-IgG | Health | 2/26 | 7.7% |
|  | IFV | 2/16 | 12.5% |
|  | NAbs Negative | 49/95 | 51.6% |
|  | NAbs Positive | 129/131 | 98.5% |
| S1-IgG | Health | 5/26 | 19.2% |
|  | IFV | 2/16 | 12.5% |
|  | NAbs Negative | 32/95 | 33.7% |
|  | NAbs Positive | 115/131 | 87.8% |
| S2-IgG | Health | 2/26 | 7.7% |
|  | IFV | 3/16 | 18.8% |
|  | NAbs Negative | 43/95 | 45.3% |
|  | NAbs Positive | 120/131 | 91.6% |
| RBD-IgG | Health | 0/26 | 0 |
|  | IFV | 0/16 | 0 |
|  | NAbs Negative | 11/95 | 11.6% |
|  | NAbs Positive | 105/131 | 80.2% |
| S82-IgG | Health | 13/26 | 50% |
|  | IFV | 6/16 | 37.5% |
|  | NAbs Negative | 49/95 | 51.6% |
|  | NAbs Positive | 102/131 | 77.9% |

**Figure S1. Sampling information in the proteome microarray.**

**
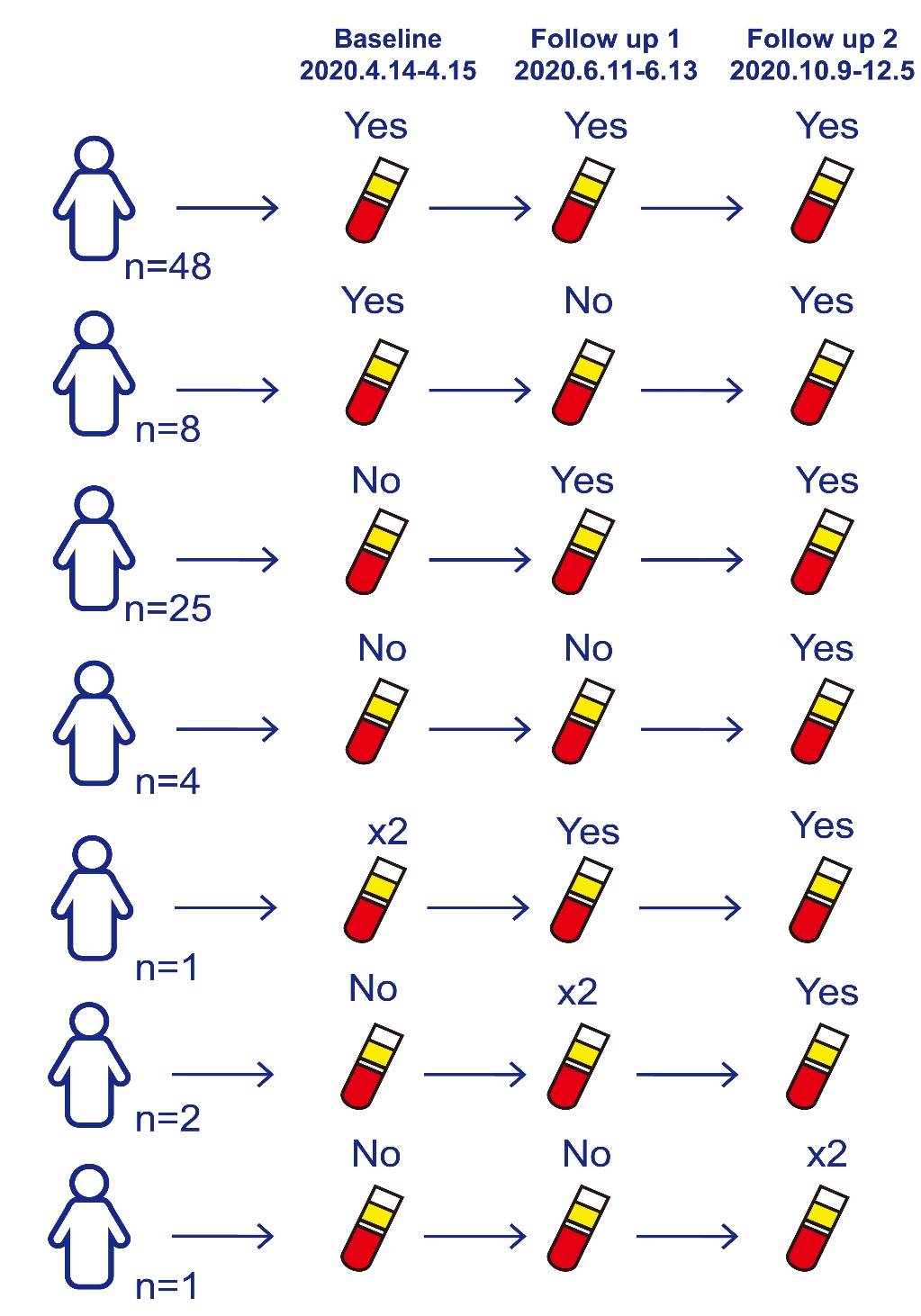
**

**Figure S2. Receiver operating characteristic (ROC) curves of NAbs evaluation using different IgGs**

**
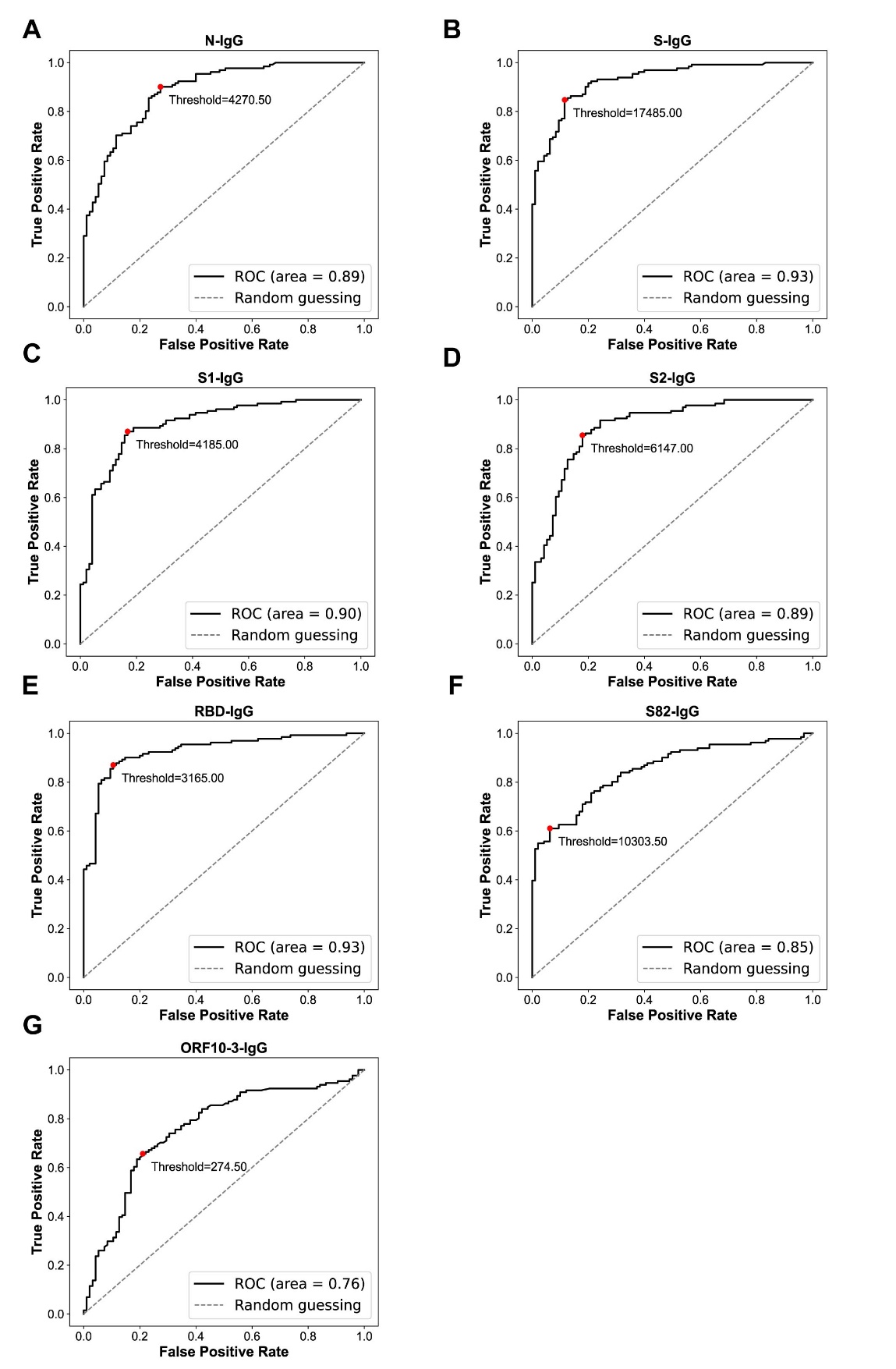
**

The X-axis indicates the false positive rate, and the Y-axis indicates the true positive rate. The red points represent the threshold of the IgG level with the highest Youden index. The areas under the curve are shown in the lower right.

**Table S3.** **The kinetic of IgG antibodies in patients with COVID-19.**

|  | P group | | | N group | | |
| --- | --- | --- | --- | --- | --- | --- |
|  | **β0** | **α0** | **FDR** | **β0** | **α0** | **FDR** |
| N-IgG | -96.09 | 32751.74 | 0.94 | -25.56 | 6456.79 | 0.99 |
| S-IgG | -44.30 | 47196.25 | 0.99 | -42.29 | 10678.28 | 0.01 |
| S1-IgG | -26.13 | 20126.69 | 0.21 | -19.43 | 5662.25 | 0.99 |
| S2-IgG | -18.15 | 20810.90 | 0.99 | -22.86 | 6262.04 | 0.86 |
| RBD-IgG | -29.86 | 19292.16 | 0.06 | -10.55 | 2449.46 | 0.02 |
| S82-IgG | -41.57 | 33758.80 | 0.65 | -16.88 | 5304.72 | 0.34 |
| ORF10-3-IgG | -0.97 | 597.02 | 0.17 | -1.19 | 312.72 | 0.003 |

α_0_ and β_0_ represent the intercept and gradient of the fixed effects in the linear mixed-effects models, which are calculated using the IgG fluorescence intensity in the microarray. FDR: False discovery rate.

**Figure S3. Information on vaccination in the vaccinated cohort.**

**
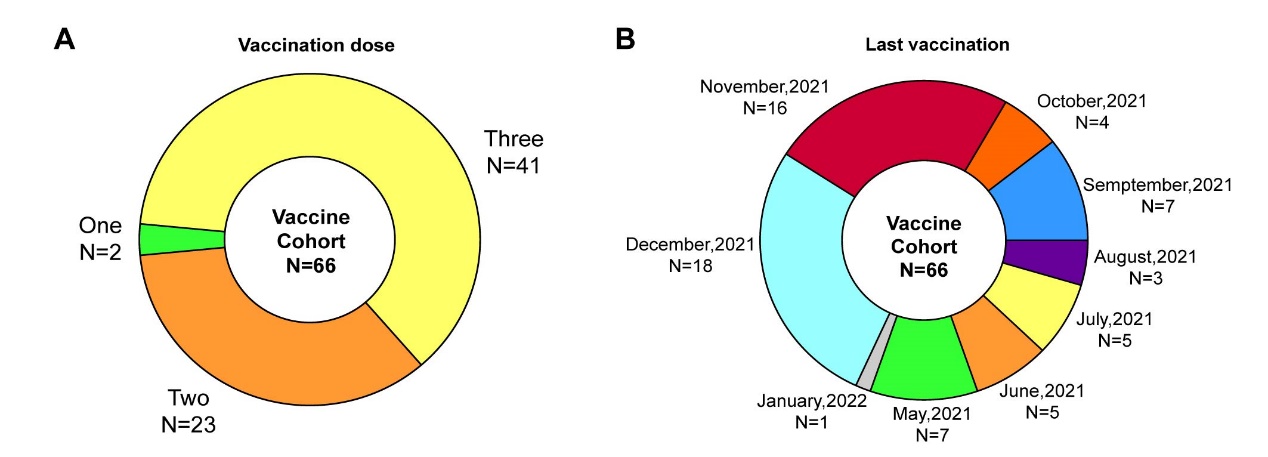
**

1. The pie diagram indicates the ratio of patients who received different vaccine doses in the cohort.
2. The time of the last vaccination for all patients in the vaccinated cohort.
